# The potential of the Child Health Utility 9D Index as an outcome measure for child dental health

**DOI:** 10.1186/1472-6831-14-90

**Published:** 2014-07-16

**Authors:** Lyndie A Foster Page, W Murray Thomson, Zoe Marshman, Katherine J Stevens

**Affiliations:** 1 Faculty of Dentistry, University of Otago, Dunedin, New Zealand; 2 Academic Unit of Dental Public Health, School of Clinical Dentistry, University of Sheffield, Sheffield, UK; 3 School of Health and Related Research (ScHARR), The University of Sheffield, Sheffield, UK

**Keywords:** Quality of life, Health utility, Dental caries, Children

## Abstract

**Background:**

The Child Health Utility 9D (CHU9D) is a relatively new generic child health-related quality of life measure (HRQoL)—designed to be completed by children—which enables the calculation of utility values.

The aim is to investigate the use of the CHU9D Index as an outcome measure for child dental health in New Zealand.

**Method:**

A survey was conducted of children aged between 6 and 9 years attending for routine dental examinations in community clinics in Dunedin (New Zealand) in 2012. The CHU9D, a HRQoL, was used, along with the Child Perceptions Questionnaire (CPQ), a validated oral health-related quality of life (OHRQoL) measure. Socio-demographic characteristics (sex, age, ethnicity and household deprivation) were recorded. Dental therapists undertook routine clinical examinations, with charting recorded for each child for decayed, missing and filled deciduous teeth (dmft) at the d_3_ level.

**Results:**

One hundred and forty 6-to-9-year-olds (50.7% female) took part in the study (93.3% participation rate). The mean d_3_mft was 2.4 (SD = 2.6; range 0 to 9). Both CHU9D and CPQ detected differences in the impact of dental caries, with scores in the expected direction: children who presented with caries had higher scores (indicating poorer OHRQoL) than those who were free of apparent caries. Children with no apparent caries had a higher mean CHU9D score than those with caries (indicating better HRQoL). The difference for the CPQ was statistically significant, but for CHU9D the difference was not significant. When the two indices were compared, there was a significant difference in mean CHU9D scores by the prevalence of CPQ and subscale impacts with children experiencing no impacts having mean CHU9D scores closer to 1.0 (representing perfect health).

**Conclusion:**

The CHU9D may be useful in dental research. Further exploration in samples with different caries experience is required. The use of the CHU9D in child oral health studies will enable the calculation of quality-adjusted life years (QALYs) for use in economic evaluation.

## Background

The biomedical model has been the dominant model of disease since the late 19^th^ century. Its emphasis is on pathological processes and the way in which they compromise the body [1]. The biomedical model has been criticised for its narrow disease-focus and failure to consider the perspective of the person affected [1,2]. More recently, there has been a shift from the biomedical to a biopsychosocial model of health [3], in which the focus is on health and illness, rather than disease. For example, dental caries is a chronic disease which affects many young children, with 50% of New Zealand 5-year-olds having experienced it [4], but relatively little research has considered the impact of dental caries on the affected children’s daily lives.

Measuring health-related quality of life (HRQoL) has been the predominant approach in research on individuals’ perspectives on their health and health care. Measures of HRQoL may be generic or specific. The former assess overall HRQoL, whereas specific measures focus on problems relevant to a specific condition, site or disease. Each is appropriate for different circumstances. Generic measures have a number of advantages. The first is their broad applicability, whereby they allow comparisons between groups or people with different conditions; second, they yield index scores that can be applied in economic evaluations of health [5]; third, they have been used more frequently than specific instruments in the general population and can be used when no specific instruments exist in a particular area. Finally, because of the broad range of aspects of health status and consequences of illness, they may detect unexpected problems associated with a condition or its treatment [6]. The main shortcoming of generic instruments relates to their broad nature, which renders them less responsive to change and less relevant or acceptable to participants with different specific conditions.

Specific instruments may be disease- or site-specific. They overcome some of the limitations of generic instruments. The relevance of their content can make them more sensitive and more acceptable to participants, and so higher completion rates are more readily achievable. Their specific nature makes them more likely to reflect true change [7]. The appropriateness of linking HRQoL to the oral cavity has been widely endorsed in the last two decades, with the development and validation of site-specific measures of oral health-related quality of life (OHRQoL) such as the Oral Health Impact Profile for adults [8] and the Child Perceptions Questionnaire CPQ; [9] for children. There has been considerable investigations comparing the validity of generic and disease specific measures in the assessment of HRQoL and OHRQoL for adults with certain measures of HRQoL (Short Form 36) found to be not as sensitive or specific as OHRQoL measures whilst others (EuroQoL and Assessment of Quality of Life (AQoL)) have shown adequate construct and convergent validity [5]. However, there has been little consideration of the measures’ application in economic evaluations of oral healthcare. Greater application has occurred in the field of medical healthcare [10], because the use of disease-specific measures as primary outcomes has been so prevalent in medicine—and because generic measures of HRQoL are often not included in investigations.—the indirect strategy (that is, mapping treatment effects on disease-specific measures to treatment effects on generic HRQoL scales; [11] is routinely used in cost-effectiveness analysis [10].

The use of economic evaluation to aid health care resource allocation decision-making is increasing internationally, particularly since the inception of national decision-making bodies such as the National Institute for Health and Care Excellence (NICE) in the UK (http://www.nice.org.uk/), the Pharmaceutical Benefits Advisory Committee (PBAC) in Australia (http://www.pbs.gov.au/info/industry/listing/participants/pbac), and PHARMAC in New Zealand (http://www.pharmac.health.nz/). Economic evaluation can assist decision-makers by providing information on the relative costs and benefits of competing alternatives. The most commonly required form of analysis is cost-utility analysis (CUA), in which the unit of benefit measurement is typically the quality-adjusted life year (QALY). The QALY combines length of life and quality of life into a single measure which is useful for decision-making because it allows for comparison both within and across different clinical domains. When combined with information on the relative costs of alternatives, the findings can be presented as incremental cost-effectiveness ratios, which allow decision-makers to view the cost-effectiveness of competing alternatives. To calculate QALYs, information is needed on individuals’ quality of life. This is commonly undertaken by the use of off-the-shelf, preference-based measures of HRQoL [11]. These consist of a questionnaire (descriptive system) for completion by the individual, along with a pre-existing set of preference weights (utility values), which are assigned to every health state defined by the descriptive system. The resulting utility values can then be combined with length of life to calculate QALYs. The use of economic evaluations in dental care has been limited principally to cost-effectiveness analyses of preventive programmes for caries [12], prosthodontics [13] and periodontal treatment [14], and very few studies have included CUA [15,16].

There are several off-the-shelf instruments available for use in adults but, until recently, instruments for paediatric QALY measurement were lacking [17]. The Child Health Utility 9D (CHU9D) is a relatively new generic HRQoL measure designed to be completed by children aged between 7 and 17 years. It enables the calculation of utility values. Children were involved throughout its development in order to ensure that it is child-centered [17]. To our knowledge, no QALY measures have yet been used with children with dental caries; if the CHU9D proved reliable and valid in this respect, the findings would support its use as an outcome measure in dental research (such as clinical trials), which would then enable CUA to be used in healthcare resource allocation decisions.

The aim of this study was to investigate the use of CHU9D as an outcome measure in this population and examine the performance of the CHU9D relative to the CPQ in New Zealand children attending for dental examinations.

## Method

A survey was conducted of approximately 150 6-to-9-year-old children attending for routine dental examinations in Dunedin community clinics in 2012. Assuming a 67% participation rate, the required sample size was estimated at 150 in order to achieve the 100 that is the minimum suggested for testing of these measures [18]. Ethical approval was obtained from the Southern Regional Ethics Committee. Written consent and assent respectively was obtained from the parent and child before proceeding. Information was gathered on each child’s sex, age and ethnicity. The children were categorised into two age groups, with “older” being the 8- and 9-year-olds, and “younger” being the 6- and 7-year-olds. An area-based deprivation measure [19] was used to allocate each participant to a deprivation decile score, based on the residential address. Areas with scores 1 to 3 were classified as “low deprivation”; those with scores 8 to 10 were classified as “high deprivation”. Two dental therapists undertook routine clinical examinations, having been trained and calibrated by an experienced examiner in the study protocol at the community clinics. No examiner reliability was undertaken. Baseline charting recorded for each child included decayed, missing and filled deciduous teeth (dmft) at the d_3_ level (clinically detectable lesion into dentine).

HRQoL was measured using the CHU9D [20], which is designed to enable the calculation of QALYs. It consists of nine dimensions (variously: worried, sad, pain, tired, annoyed, schoolwork/homework, sleep, daily routine and activities), each represented by a single question with five response options. The recall period is today/last night, and the questionnaire is completed by the child (where possible). The responses to each of the nine questions can be taken together as a description of the HRQoL of the child; this is termed a “health state”. There are many different health states defined by the CHU9D descriptive system (due to the different combinations of response options on each of the nine dimensions), and each unique health state has a preference weight associated with it. These preference weights give a utility value (on a 0–1 scale where 1 is perfect health and 0 is a state equivalent to being dead) which, when combined with length of life, enables the calculation of QALYs [20]. To calculate QALYS for the caries and not apparent caries groups in this sample, we assumed that we are looking at the childhood period from age six to eighteen years (a total of 12 years). We took the mean utility value for each group and multiplied it by 12 (years) to give mean QALYs for both groups. We assumed no discounting as the QALY calculation here is for illustrative purposes. We then subtracted these to give the difference in mean QALYs.

To examine the performance of the CHU9D, childrens’ perceptions of the impact of their oral health were measured using a validated OHRQoL measure, the 16-item short-form CPQ-ISF:16 (CPQ) questionnaire previously validated in this age-group [21]. The research assistant administered the questionnaire, reading each question to the child. Response options and scores were: ‘Never’ (scoring 0); ‘once or twice’ [1]; ‘Sometimes’ [2]; ‘Often’ [3]; and ‘Every day or almost every day’ [4]. At the analysis stage, an overall CPQ score was computed by summing the appropriate item scores for each measure, with a higher score indicating poorer OHRQoL. The prevalence of one or more impacts was determined by identifying those who responded ‘Often’ or ‘Every day or almost every day’ for at least one of the 16 CPQ items.

Childrens’ perceptions of their oral health were also assessed using two global measures. First, they were asked to rate the health of their teeth and mouth (response options: ‘Very good,’ ‘Good,’ ‘OK’ or ‘Poor’). Second, they were asked how much their teeth or mouth impacted on their life overall (response options: ‘Not at all,’ ‘A little bit,’ ‘Some’ or ‘A lot’). For the first, responses to these were dichotomised to enable comparison of those rating their oral health ‘Very good’ or ‘Good’ with the remainder; for the second, responses were dichotomised to enable comparison of those who responded “Not at all” with the remainder.

Data were analysed with SPSS (version 20.0). A score of zero was used where responses to questions were missing. The computation of descriptive statistics was followed by bivariate analyses, which used Chi-square tests for comparing proportions; Mann–Whitney or Kruskal–Wallis tests were used (as appropriate) for comparing scores for continuous variables (where these were not normally distributed). Where continuous variables were normally distributed, ANOVA was used to compare means. The alpha value was set at 0.05. Pre-existing preference weights were applied to the responses to the CHU9D questions in order to calculate the utility values. These preference weights are based on a UK survey of general population values, using the standard gamble method [20].

## Results

One hundred and forty 6-to-9-year-olds (50.7% female) took part in the study (93.3% participation rate). Nearly all were nonMāori (Table 1), and approximately one-quarter resided in highly deprived areas. The overall mean d_3_mft was 2.4, and scores ranged from 0 to 9. Nearly two-thirds (62.4%) had experienced caries (dmft > 1).

**Table 1:** Sociodemographic characteristics and dental caries status

**Characteristics**	**N**	**No apparent caries (% dmft = 0)**	**Mean dmft (SD)**
All	140	54 (38.6)	2.4 (2.6)
Sex			
Male	69	28 (40.6)	2.3 (2.6)
Female	71	26 (36.6)	2.6 (2.7)
Age (years)			
6-7	50	23 (46.0)	2.0 (2.6)
8-9	90	31 (34.4)	2.7 (2.7)
Ethnicity			
NonMāori	123	49 (39.8)	2.3 (2.5)
Māori	17	5 (29.4)	3.8 (3.1)
Deprivation			
High	36	11 (30.6)	3.1 (2.9)
Medium	58	28 (48.3)	2.1 (2.6)
Low	45	15 (33.3)	2.3 (2.4)

The mean score for the CHU9D was 0.87 (SD = 0.10), with scores ranging from 0.56 to 1.00, where 1 is perfect health. The overall mean CPQ score was 11.7 (SD = 9.2; range 0 to 47), and scores were positively skewed. Floor-effects were non-existent for the CHU9D and almost non-existent for the CPQ, with (respectively) 0.0% and 0.7% of children having zero scores. Ceiling effects for both measures were small, with 9.3% and 0.7% having the maximum score of 1 and 47, for the CHU9D and CPQ respectively. The only missing values were found in the CPQ questionnaire, where two oral symptoms items and one emotional well-being item were not completed.

Children who presented with caries had lower CHU9D and higher CPQ scores (indicating poorer HRQoL and OHRQoL) than those who did not have apparent caries. The difference in CPQ score between those with or without apparent caries was statistically significant (Table 2). There was a significant difference in the mean CHU9D scores by the prevalence of CPQ_11–14_ and subscale impacts, with children experiencing no impacts having mean CHU9D scores closer to 1.0 (Table 3).

**Table 2:** **Mean CHU9D and CPQ**_**11–14 **_**(and subscale) scores by sociodemographic characteristics and caries experience**

	**CHU9D (SD)**	**CPQ**_**11–14 **_**(SD)**				
		**Overall score**	**Subscales**			
			**Oral symptoms**	**Functional limitations**	**Emotional well-being**	**Social well-being**
All children						
Age 6 to 7	0.87 (0.10)	12.4 (7.5)	4.7 (2.5)	4.1 (2.8)^a^	2.0 (2.1)	1.8 (2.6)
Age 8 to 9	0.87 (0.10)	11.3 (10.0)	4.3 (3.0)	3.2 (3.0)	2.5 (3.3)	1.3 (2.3)
Sex						
Male	0.86 (0.10)	11.8 (9.5)	4.4 (2.7)	3.6 (2.9)	2.5 (3.2)	1.6 (2.6)
Female	0.88 (0.10)	11.6 (8.9)	4.5 (2.9)	3.6 (3.0)	2.1 (2.6)	1.4 (2.2)
Ethnicity						
NonMāori	0.87 (0.10)	11.3 (9.1)^a^	4.3 (2.8)	3.5 (3.0)	2.1 (2.7)	1.5 (2.4)
Māori	0.87 (0.10)	15.1 (9.5)	5.5 (2.9)	3.9 (2.7)	3.5 (3.8)	1.8 (2.5)
NZDep						
High	0.86 (0.10)	13.4 (10.2)	4.9 (3.1)	4.2 (3.2)	2.9 (3.4)	1.8 (2.9)
Medium	0.88 (0.10)	10.6 (8.0)	4.3 (2.6)	3.1 (2.7)	1.9 (2.6)	1.2 (2.0)
Low	0.87 (0.09)	12.1 (9.8)	4.4 (2.9)	3.7 (3.0)	2.3 (2.9)	1.7 (2.5)
Caries prevalence						
No apparent caries (dmft = 0)	0.88 (0.10)	9.7 (9.0)^a^	4.1 (2.9)	3.1 (2.9)	1.6 (2.6)^a^	0.9 (1.9)^a^
Caries (dmft > 0)	0.87 (0.10)	13.0 (9.1)	4.7 (2.7)	3.8 (3.0)	2.7 (3.0)	1.9 (2.7)

^a^P < 0.05; Kruskal-Wallis/Mann–Whitney.

**Table 3:** **Mean CHU9D by CPQ**_**11–14 **_**impact prevalence**

	**CHU9D (SD)**	**P value**
Overall CPQ_11–14_ score		
No impacts	0.90 (0.08)	0.002
1+ impacts	0.85 (0.11)	
Oral symptoms		
No impacts	0.89 (0.09)	0.019
1+ impacts	0.85 (0.10)	
Functional limitations		
No impacts	0.89 (0.09)	<0.001
1+ impacts	0.82 (0.11)	
Emotional well-being		
No impacts	0.89 (0.09)	<0.001
1+ impacts	0.79 (0.11)	
Social well-being		
No impacts	0.88 (0.09)	0.001
1+ impacts	0.80 (0.11)	

There were statistically significant differences in scores between those reporting no impact on their life overall and those reporting some impact as shown in Table 4. Both the CHU9D and CPQ demonstrated statistically significant correlations with impact on life overall. The magnitude of the correlations was similar but the signs were opposite as higher impact scores indicate a higher impact of problems while higher utility scores reflect better health states. The CHU9D was found to be reliable, with a Cronbach’s alpha score of 0.66; that for the CPQ was 0.86.

**Table 4:** Mean CPQ and CHUD9 scores by global oral health questions

	**CPQ**_**11–14 **_**(SD)**	**CHU9D (SD)**
Self-rated oral health		
Very good (N = 28)	9.9 (9.7)	0.87 (0.12)
Good/Fair/Poor (N = 112)	12.2 (9.0)	0.87 (0.09)
Spearmans rho	0.15	- 0.06
Impact of oral health on quality of life		
Not at all (N = 53)	8.6 (7.6)	0.91 (0.10)
A little bit/Some/A lot (N = 87)	13.6 (9.6)^b^	0.85 (0.09)^a^
Spearmans rho	0.30^c^	- 0.38^c^

^a^P < 0.01 Kruskal-Wallis.

^b^P < 0.05 Kruskal-Wallis.

^c^Correlation significant at 0.01 level.

The mean QALYs for children with apparent caries was 10.56; for the no apparent caries group, it was 10.44. The difference in QALYs between those with no apparent caries and those with caries was 0.12.

## Discussion

The use of economic evaluation to aid health care resource allocation decision-making is increasing internationally, although there has been little application of utility measures in economic evaluations of paediatric oral healthcare to date. The CHU9D shows promise as an outcome measure for this purpose when examining its performance with the CPQ in children attending for a dental examination in New Zealand. The use of the CHU9D in future child oral health studies will enable the calculation of quality-adjusted life years (QALYs) for use in economic evaluation.

The findings showed that the child OHRQoL measure (the CPQ) was more sensitive to the oral health variable ‘caries’ in the current study than the generic HRQoL measure, the CHU9D. This is in keeping with findings from adult populations examining the use of generic and disease specific measures of oral health, where the Oral Health Impact Profile was found to be more sensitive to oral health variables than two generic measures, the EQ-5D and the AQoL [5]. However, the CHU9D did demonstrate an association, indicating adequate construct validity. The mean CHU9D utility value found in the current study is consistent with other recent studies. In a community sample of Australian 11-17-year-olds, the mean CHU9D utility was 0.85 [22], while it was found to be 0.86 in a UK school-based sample of 6–7 year olds [23]. The mean utility value for the group without caries in the New Zealand sample was similar to those observed other populations. While the CHU9D did show a difference in utility score in the expected direction, the difference was not significant, indicating that the CHU9D may not be able to detect the impacts of dental caries, either because the impacts were too low or the CHU9D was not sensitive enough in this sample. Other studies using the CPQ in children with low caries experience have also found a tenuous association with clinical indicators [24,25]. However, there was a significant difference in the mean CHU9D scores by the prevalence of CPQ and subscale impacts, with children experiencing no impacts having mean CHU9D scores closer to 1.0, indicating that the CHU9D has good criterion validity in comparison to the CPQ.

That both the CPQ and CHU9D were associated with the rating of impact on life overall further supports the usefulness of the CHU9D, in that the observed gradients in mean scores across the categories of that global item show that its concurrent validity was excellent. However, this was not the case for the global rating of oral health. It has been found that, generally, there is a stronger relationship between ratings of the impact on life overall than self-rated oral health in children of this age [21]. The internal consistency reliability of the CHU9D was found to be lower than that of the CPQ and just below the arbitrary threshold Cronbach’s alpha value for sufficient internal consistency of 0.7. [26]. The relatively low value for the CHU9D is probably due to its lower number of items [26]. Utility measures typically only include a small number of items and this is particularly the case for the CHU9D which was designed to minimise the response burden on child participants.

Translating the utility scores into QALYs allows comparison of the benefits with other clinical areas. QALYs are particularly useful in cost utility analysis because, when combined with information on the costs of alternatives, incremental cost-effectiveness ratios (ICER) can be calculated. The ICERs can then be compared both within and across clinical areas and provide important information on the relative cost-effectiveness of interventions. For example, a recent paper [16] compared the cost-effectiveness of two different caries prevention programmes, with cost-effectiveness expressed in terms of the cost per prevented DMF surface. While this is useful information, it does not allow comparison with other programmes (apart from within dentistry, where DMFS is widely used and understood). As part of their trial, Vermaine et al. undertook a micro costing analysis of the prevention programmes and found that, from a health care perspective, the incremental cost of an increased professional fluoride application (IPFA) programme over standard dental care was €37 over three years (undiscounted). As an illustration, our data on QALYs could be combined with this to calculate an ICER. Let us assume that we have two groups, one receiving standard dental care and the other receiving IPFA over a period of 12 years. The cost for 12 years (multiplying the incremental 3-year cost by 4)—assuming no discounting—would give an incremental cost of €148. If we assume that the IPFA programme is 100% effective in preventing caries, then, using our utility data, we would have a difference in QALYs over the 12 years of 0.12 (this assumes that our standard dental care group would have caries and the IPFA group no caries). The ICER would therefore be €148/0.12 = €1,233. While this calculation is based on strong assumptions, it is provided for illustrative purposes only, to demonstrate the usefulness of having the cost-effectiveness of interventions expressed as an ICER. Future research should concentrate on collecting cost and utility data in order to allow the economic evaluation of dental interventions and hence lead to more effective and efficient health care resource allocation decision-making.

The high participation rate of the current study (with nearly all the children invited having consent and assenting to take part) is a major strength, since 100 participants is the minimum suggested for testing of these measures [18]. Among the study’s other strengths was that the children self-reported their own responses to both measures. The value of getting children to express their opinions about their oral health is becoming increasingly important in dentistry [27]. There has been wider recognition that, since it is the child who receives the treatment and lives with the consequences, his/her opinions are important and credible [28], but it is only recently that dentistry has begun to seek such information from children. The CHU9D was developed entirely with children, from a series of interviews with children aged 7–11 years and is very child–focused as a result.

A main limitation of this study was that the children represented a convenience sample of participants who were attending for routine examinations; hence, the findings may not be generalisable. Children in this region have some of the lowest caries experience in New Zealand, with a mean dmft of 1.3 for five-year-olds, whereas the overall mean dmft for this age group in New Zealand is 2.4. However, comparisons between the study findings and national data should be made with caution as the children in this study were older than five years and the national data are collected based on different criteria [29].

## Conclusion

In conclusion, the CHU9D may be useful in dental research. Further exploration in samples with different caries experience is required. The use of the CHU9D in child oral health studies will enable the calculation of quality-adjusted life years (QALYs) for use in economic evaluation.

## Competing interests

The authors declare that they have no competing interests.

## Authors’ contributions

LFP, KS, ZM and WMT all were involved in the conceptualization of the study. LFP collected the data using the questionnaire developed by KS. KS and ZM contributed to the analytical strategy with WMT advising on different methodological approaches to the data. LFP drafted the paper with substantive contribution from ZM, KS and WMT. All authors read and approved the final manuscript.

## Pre-publication history

The pre-publication history for this paper can be accessed here:

http://www.biomedcentral.com/1472-6831/14/90/prepub
